# COVID-19 pandemic and waiting times in outpatient specialist care in Germany: an empirical analysis

**DOI:** 10.1186/s12913-021-07094-9

**Published:** 2021-10-11

**Authors:** Jennifer Muschol, Christian Gissel

**Affiliations:** Department of Health Economics, Justus Liebig University, Giessen, Germany

**Keywords:** COVID-19, Corona, Waiting time, Outpatient, Specialist, Health service, Healthcare access, Inequalities

## Abstract

**Background:**

International healthcare systems face the challenge that waiting times may create barriers to accessing medical care, and that those barriers are unequally distributed between different patient groups. The disruption of healthcare systems caused by the COVID-19 pandemic could exacerbate this already strained demand situation. Using the German healthcare system as an example, this study aims to analyze potential effects of the COVID-19 pandemic on waiting times for outpatient specialist care and to evaluate differences between individual patient groups based on their respective insurance status and the level of supply.

**Methods:**

We conducted an experiment in which we requested appointments by telephone for different insurance statuses in regions with varying levels of supply from 908 outpatient specialist practices in Germany before and during the COVID-19 pandemic. Data from 589 collected appointments were analyzed using a linear mixed effect model.

**Results:**

The data analysis revealed two main counteracting effects. First, the average waiting time has decreased for both patients with statutory (mandatory public health insurance) and private health insurance. Inequalities in access to healthcare, however, remained and were based on patients’ insurance status and the regional level of supply. Second, the probability of not receiving an appointment at all significantly increased during the pandemic.

**Conclusions:**

Patient uncertainty due to the fear of a potential COVID-19 infection may have freed up capacities in physicians’ practices, resulting in a reduction of waiting times. At the same time, the exceptional situation caused by the pandemic may have led to uncertainty among physicians, who might thus have allocated appointments less frequently. To avoid worse health outcomes in the long term due to a lack of physician visits, policymakers and healthcare providers should focus more on regular care in the current COVID-19 pandemic.

## Background

Waiting times are serious challenges for international healthcare systems. Since they affect patients’ access to medical care, international institutions such as the Organisation for Economic Co-operation and Development (OECD) are addressing this issue [1].

Waiting times can negatively affect perceived satisfaction on the demand side by delaying treatments for patients, thus extending their state of suffering and leading to uncertainty [2, 3]. In addition to declining patient satisfaction, however, the potential risk that waiting times lead to inferior health outcomes is a cause for concern. Study results show an association between increased waiting times and poorer health outcomes for some conditions such as psychosis, increased hospitalizations for ambulatory care sensitive conditions, and higher mortality [4–6].

Unequal access to care for specific groups is an additional challenge in many healthcare systems. International studies have analyzed this problem using various approaches. It has been shown that patient characteristics such as income, education and socioeconomic status can have a significant effect on the extent of waiting times and access to healthcare, resulting in inequalities. These effects are apparent in both inpatient [2, 7–10] and outpatient care [11, 12].

In addition to these factors, the insurance status of patients can also lead to unequal access to healthcare [13]. Although the distribution of waiting times is a significant component of everyday care for patients, empirical evaluations of the impact of insurance statuses on waiting times are limited in current research [14]. Few studies have so far addressed this issue in depth.

Germany, with its two-tier insurance system, provides a suitable framework for analyses of differences between insurance statuses [15, 16]. Health insurance is mandatory in Germany and people are either covered by statutory health insurance (SHI) or by private health insurance (PHI). In 2021, around 89% of patients are covered by SHI, whereas 11% of the population are insured by PHI [17]. German SHI is a public scheme that is mandatory for the majority of the population. Only a minority of the population is exempt from SHI and can voluntarily take out PHI. SHI premiums are based on the income of the insured, whereas PHI premiums are risk-based [18]. Any unequal treatment of these two insurance groups may largely be due to the fact that physicians can charge medical interventions for PHI patients both more frequently and at higher rates [19]. The resulting financial incentive for physicians to treat PHI patients preferentially [15] has been evaluated in a small number of studies for both inpatient and outpatient settings in Germany. These studies show that PHI patients receive earlier access to care than SHI patients due to faster appointment scheduling [14, 15, 19, 20]. Schwierz et al. also found that hospitals that discriminate based on patients’ insurance status show better financial results [20].

Three studies analyzed experimental data of German outpatient specialist care: Lungen et al. (2008) [21], Heinrich et al. (2018) [22] and Werbeck et al. (2020) [23]. Using an experimental design in which outpatient specialists were called and asked for an appointment, these studies examined determinants of waiting times with a special focus on patients’ insurance status. All three studies conclude that PHI patients receive preferential treatment regarding waiting times.

All of these observations show that difficulties arise both from waiting times themselves and from differences in waiting times between individual patient groups. As a result, international healthcare systems are trying to counter the effects resulting from a form of rationing [10] and discrimination [11] through policy interventions [1], highlighting the fact that the organization of medical supply is critical.

The global COVID-19 outbreak, which was declared a pandemic by the World Health Organization (WHO) in March 2020 [24], could exacerbate this condition. COVID-19 is disrupting healthcare systems and medical care worldwide. This could have a major impact on previously existing structures and processes on the demand and supply side of healthcare [25]. Waiting times could be one of the variables that are strongly affected by the current pandemic.

Although research on COVID-19 is strong in a variety of fields, and there is heightened awareness of the need not to neglect patients without COVID-19 [26–28], there are currently no analyses of changes in waiting times in the context of the pandemic. This is what the present analysis focuses on. We seek to analyze the potential effect of the COVID-19 pandemic on outpatient specialist care in terms of waiting time and to evaluate differences for patient groups with different insurance statuses, using the German healthcare system as an example. In addition, our study contributes to the existing literature by explicitly including the level of supply in Germany and examining it for potential inequalities. It is evident that there are structural differences and barriers to accessing healthcare in Germany. According to the German Social Law, the goal of healthcare should be to ensure that patients are treated according to their needs and in a uniform manner. Favoring a particular patient group or accepting structural differences in care is inconsistent with this guiding principle [19, 29]. In this sense, it is essential to consider the impact of the COVID-19 pandemic on an already strained demand situation.

## Methods

### Data collection

The present study was originally designed to analyze waiting times for outpatient specialist care for different patient groups based on their respective insurance status and the level of supply. However, due to the unexpected occurrence of the COVID-19 pandemic, the study objective was adapted. We therefore conducted an experiment in which we called outpatient specialist practices and asked for an appointment in order to evaluate waiting times and the impact of the COVID-19 pandemic. Data collection design is similar to that used by Lungen et al. [21], Heinrich et al. [22] and Werbeck et al. [23].

In line with the original study design, the analysis focused on general outpatient specialist care that was not directly involved in the treatment of COVID-19. Physicians of highly specialized medical fields were excluded. Surgeons and orthopedists, gynecologists, dermatologists, otorhinolaryngologists as well as ophthalmologists were contacted because they represented the most frequently practicing specialists.

Regionally, the federal state of Bavaria was examined, as the difference between over- and underserved areas was particularly pronounced there. Using data from the Bavarian Association of Statutory Health Insurance Physicians (BASHIP), the regions most affected by over- and undersupply were identified. With the help of the physician and psychotherapist search of BASHIP, the specialists of the medical specializations described above were selected in the respective regions. The first data collection took place in April 2019 and the second one in April 2020. In both years every practice was contacted by employees of the institute: once as a patient with SHI coverage and once as a patient with PHI coverage. The insurance status was randomized. The aim was to analyze potential differences in waiting times based on patients’ insurance status.

On the basis of a given protocol, the practices were called and asked for the next possible appointment regardless of a specific physician. The reasons given for the examination were an eye examination, back pain, a gynecological examination, a hearing test and screening for skin cancer. These are, among others, the most common reasons for treatments by the respective specialists. All queries avoided giving the impression of an emergency. Each appointment was rejected at the end of the call to ensure that resources were protected. The time between the call and the first possible appointment was recorded. This number of days was adjusted for weekends and public holidays. Thus, the waiting time considered represents the number of weekdays. If no appointment was assigned, it was noted with a reason. Missing data for practices that were newly opened or closed in 2020 were also noted. Due to the repeated measurements and partially missing data, the observation represents an unbalanced panel.

### Statistical methods

In a first step, results are presented as mean, standard deviation and median. Univariate statistics are considered to measure the influence of the COVID-19 pandemic on waiting times. The measurements (longitudinal data) are correlated because they were obtained from the same practices. Since waiting times are not normally distributed and observations are dependent, the non-parametric Wilcoxon signed-rank test was used to compare the parameters between 2019 and 2020. Pearson’s Chi-squared test was applied to examine differences in the likelihood of receiving an appointment between insurance statuses. Because of multiple testing, the significance levels were adjusted by a Bonferroni-Holm correction.

Furthermore, in the context of the subsequent multivariate analysis, a linear mixed effect model (LMEM) was applied. LMEMs contain fixed effects that allow a systematic time trend component in the average response to be modelled to assess systematic differences between parameters with respect to the time trend. In addition, they take into account the correlation structure within practices and the random variation in time between practices on the basis of random effects.

Using the maximum-likelihood estimation procedure, a large number of differently constructed models were computed and compared based on the Akaike information criterion (AIC). The purpose was to find the best possible model for the data collected.

The computation of the intraclass correlation coefficient (ICC) with a random intercept only model revealed evidence of substantial clustering, where 42.4% of the variation in waiting times resulted from differences between practices. The variation in the random intercept was statistically significant. We included random intercepts for the practices as well as random slopes for the effect of year for the practices. Missing values were considered to be missing at random (MAR) because they do not depend on the waiting time itself.

Because of the non-normal distribution, the dependent variable waiting time was logarithmized. A simple and an extended model were used to analyze the data. The following equation reflects the simple model:

ln(WT)_ij_ = (ß_0_ + u_0j_) + (ß_1_ + u_1j_)*YEAR_ij_ + ß_2_*SPEC_ij_ + ß_3_*INS_ij_ + ß_4_*SUP_ij_ + ε_ij_.

As fixed effects, we entered year, medical specialization, insurance status and level of supply.

Year is coded as a dummy variable for 2019 and 2020. The categorial variable medical specializations contains surgeons and orthopedists, gynecologists, dermatologists, otorhinolaryngologists and the reference group ophthalmologists. The remaining two fixed effects are also coded as dummies. PHI and SHI determine the insurance status, and the level of supply consists of oversupply and undersupply.

ln(WT)_ij_ = (ß_0_ + u_0j_) + (ß_1_ + u_1j_)*YEAR_ij_ + ß_2_*SPEC_ij_ + ß_3_*INS_ij_ + ß_4_*SUP_ij_ + ß_5_*WEEKD_ij_ + ß_6_*DAYT_ij_ + ß_7_*(YEAR_ij_*SPEC_ij_) + ε_ij_.

The extended model includes all effects of the simple model, despite a control for the fixed effects weekday of call categorized for the workdays Monday to Friday, with Friday as reference category and the dummy variable daytime of call comprising morning and afternoon. Additionally, an interaction effect between year and medical specialization was added. Visual inspection of residual plots for both models did not reveal any obvious deviations from linearity, homoscedasticity or normality.

## Results

There were *n* = 213 practices in 2019 and *n* = 241 practices in 2020 that were contacted twice. The number of practices changed because some practices were closed and others were reopened. Thus, a total of 908 observations were collected, which resulted in 598 appointments. The inclusion rate (percentage of appointments received from practices contacted) was 63.38% for SHI in 2019, 72.30% for PHI in 2019, 57.26% for SHI in 2020, and 67.22% for PHI in 2020, with an overall inclusion rate of 64.87%.

The following reasons resulted in no appointment being set up. In 27.3% of the cases, the practice did not treat the inquired indication; 18.41% of practices were newly opened or closed within both years; 13.30% did not accept new patients; in 13.30% of cases the practices could not be reached by telephone; 13.30% of appointments were declined due to the COVID-19 pandemic; 11.25% of practices were on vacation or temporarily closed; finally, 3.07% of requests were denied because a referral was required or the practice could be visited without an appointment.

Table 1 shows the mean, standard deviation and median as well as the comparison of the parameters between 2019 and 2020 with help of the Wilcoxon signed-rank test. There are strong deviations between the mean and the median within the groups as well as considerable differences in the mean waiting time between the individual groups.

**Table.1:** Test statistic – Wilcoxon signed-rank test

Variables	n	Mean	SD	Median	Variables	n	Mean	SD	Median	z	p	r
Waiting time 19 SHI	135	26.13	29.55	15	Waiting time 20 SHI	138	17.09	30.48	5	−4.33	< .001	.43
Waiting time 19 PHI	154	17.37	20.25	12	Waiting time 20 PHI	162	11.09	18.83	4	−4.01	< .001	.36
Waiting time 19 SHI	135	26.13	29.55	15	Waiting time 19 PHI	154	17.37	20.25	12	−5.78	< .001	.50
Waiting time 20 SHI	138	17.09	30.48	5	Waiting time 20 PHI	162	11.09	18.83	4	−4.04	< .001	.36
Difference 19	132	10.40	23.75	2	Difference 20	129	5.76	22.14	1	−2.20	.028	.22
Waiting time undersupply 19	43	24.78	21.31	17	Waiting time undersupply 20	41	18.57	29.55	6.5	−2.94	.003	.54
Waiting time oversupply 19	89	18.52	21.67	10.5	Waiting time oversupply 20	88	8.93	10.33	4	−3.75	< .001	.46
Waiting time Surgeons & Orthopedists 19	38	10.79	9.30	7	Waiting time Surgeons & Orthopedists 20	40	4.34	4.98	3	−4.71	< .001	.86
Waiting time Gynecologists 19	39	23.59	28.53	15	Waiting time Gynecologists 20	38	20.30	18.34	15.25	−.97	.330	.19
Waiting time Dermatologists 19	14	34.40	17.45	31	Waiting time Dermatologists 20	8	12.75	9.27	12.25	−2.02	.043	.90
Waiting time Otorhinolaryngologists 19	23	18.63	20.35	9	Waiting time Otorhinolaryngologists 20	26	3.88	2.85	3.25	−3.96	< .001	.87
Waiting time Ophthalmologists 19	18	26.31	20.30	21.75	Waiting time Ophthalmologists 20	17	23.50	38.45	12.5	−1.78	.075	.49

The differences between the mean and median and the large standard deviation can be explained by strong outliers in the data set. For instance, the maximum waiting time is 162 days for SHI 2019, 120 days for PHI 2019, 259 days for SHI 2020, and 122 for PHI 2020. The graphical illustration of the waiting times showed that the data are not normally distributed; they are right skewed as well as leptokurtic.

The overview reveals that the longest mean waiting time, at 26.13 days, was faced by SHI patients in 2019. PHI patients had to wait approximately nine days less, resulting in a mean of 17.37 days. For both insurance statuses, the waiting times decreased in 2020 by an overall average of almost 8 days or exactly 35.2% with 17.09 days for SHI patients and 11.09 days for PHI patients.

The Wilcoxon signed-rank test reveals significant differences in the comparison of the groups. The waiting time for SHI patients is significantly lower in 2020 than in 2019 (z = − 4.33, *p* = <.001, *n* = 102, r = .43). The same applies for PHI patients (z = − 4.01, *p* = <.001, *n* = 124, r = .36). Simultaneously, the findings of the observation support the already existing literature [21–23]. Both in 2019 (z = − 5.78, *p* = <.001, *n* = 132, r = .50) and in 2020 (z = − 4.04, *p* = <.001, *n* = 129, r = .36) SHI patients had to face significantly longer waiting times than PHI patients. According to Cohen’s effect size, 2019 shows a strong effect. However, it can also be seen that the overall difference in waiting time between SHI and PHI patients became significantly smaller in 2020 with a mean of 10.40 to 5.76 days (z = − 2.20, *p* = .028, *n* = 96, r = .22). The change between the two years is also reflected in the level of supply. The average value between SHI and PHI patients was considered for under- and oversupplied regions. The waiting times decreased from 24.78 in 2019 to 18.57 days in 2020 (z = − 2.94, *p* = .003, *n* = 30, r = .54) in regions with undersupply and from 18.52 to 8.93 days (z = − 3.75, *p* = <.001, *n* = 66, r = .46) in regions with oversupply. In this context, it is noticeable that patients in underserved regions face higher waiting times than those in overserved regions. Considerable differences are also apparent when considering the medical specializations. The average waiting time for SHI and PHI patients decreased for all specializations. While the results for gynecologists and ophthalmologists are not statistically different from zero, the mean waiting times for surgeons and orthopedists decreased significantly by 6.45 days (z = − 4.71, *p* = <.001, *n* = 30, r = .86), for dermatologists by 21.65 days (z = − 2.02, *p* = .043, *n* = 5, r = .90) and for otorhinolaryngologists by 14.75 days (z = − 3.96, *p* = <.001, *n* = 21, r = .87) in 2020. This represents strong effects according to Cohen’s d. Thus, it appears that waiting times decreased for all parameters due to the COVID-19 pandemic.

It should be noted, however, that the result for dermatologists and the difference between the insurance statuses 2019 and 2020 are no longer statistically different from zero after a Bonferroni-Holm correction.

In addition to considering the waiting times within the groups, the probability of receiving an appointment at all was also analyzed using Pearson’s chi-square. To avoid bias, only two reasons that practices offered for not making assignments were considered here: not accepting new patients and not assigning appointments due to the COVID-19 pandemic. The results can be found in Table 2.

**Table.2:** Test statistic – Pearson’s chi-square

Variables	Probability	Variables	Probability	χ^2^	df	p	n	V
Appointment 19	89.8%	Appointment 20	80.9%	10.68	1	.001	693	.124
Appointment SHI 19	84.4%	Appointment SHI 20	74.6%	4.97	1	.026	345	.120
Appointment PHI 19	95.1%	Appointment PHI 20	87.1%	6.58	1	.010	348	.137
Appointment SHI both years	79.1%	Appointment PHI both years	90.8%	18.51	1	< .001	693	.163
Appointment SHI 19	84.4%	Appointment PHI 19	95.1%	10.00	1	.002	322	.176
Appointment SHI 20	74.6%	Appointment PHI 20	87.1%	9.37	1	.002	371	.159
Undersupply 19	87.3%	Undersupply 20	75.8%	5.10	1	.024	238	.146
Oversupply 19	91.0%	Oversupply 20	83.5%	5.64	1	.018	455	.111
Surgeons & Orthopedists 19	96.3%	Surgeons & Orthopedists 20	95.6%	.05	1	.820	170	.017
Gynecologists 19	86.3%	Gynecologists 20	73.6%	5.50	1	.019	227	.156
Dermatologists 19	96.8%	Dermatologists 20	69.4%	8.46	1	.004	67	.355
Otorhinolaryngologists 19	100%	Otorhinolaryngologists 20	98.2%	.86	1	.353	102	.092
Ophthalmologists 19	75.8%	Ophthalmologists 20	66.2%	1.43	1	.231	127	.106

The analysis shows that the probability of receiving an appointment decreased significantly in 2020 (80.9%) compared to 2019 (89.8%) (χ^2^(1) = 10.68, *p* = .001, *n* = 693, V = .124). The lower likelihood of receiving an appointment due to the COVID-19 pandemic is also reflected in the segmentation between the insurance statuses. SHI patients received significantly fewer appointments when comparing 2019 (84.4%) to 2020 (74.6%). (χ^2^(1) = 4.97, *p* = .026, *n* = 345, V = .120). Also, PHI patients received appointments less frequently between 2019 (95.1%) and 2020 (87.1%) (χ^2^(1) = 6.58, *p* = .010, *n* = 348, V = .137). At the same time, unequal access to healthcare is also evident in the likelihood of receiving an appointment. Thus, SHI patients (79.1%) were significantly less likely to receive an appointment than PHI patients (90.08%) in both years (χ^2^(1) = 18.51, *p* < .001, *n* = 693, V = .163). This is also reflected in the analysis of the two years. PHI patients were more likely to receive appointments both in 2019 (95.1%) and in 2020 (87.1%) than SHI patients (2019: 84.4%, 2020: 74.6%) (2019: (χ^2^(1) = 10.00, *p* = .002, *n* = 322, V = .176); 2020: (χ^2^(1) = 9.37, p = .002, *n* = 371, V = .159)).

The decreased likelihood of receiving an appointment in 2020 is also evident in underserved regions with 11.5% fewer appointments (χ^2^(1) = 5.10, *p* = .024, *n* = 238, V = .146) and in overserved regions with 7.5% fewer appointments (χ^2^(1) = 5.64, *p* = .018, *n* = 455, V = .111).

The probabilities decreased in all medical specializations, but the change was only significant for gynecologists, who went from 86.3% in 2019 to 73.6% in 2020 (χ^2^(1) = 5.50, *p* = .019, *n* = 227, V = .156), and for dermatologists, who went from 96.8% to 69.4% (χ^2^(1) = 8.46, *p* = .004, *n* = 67, V = .355). Again, the effects for SHI appointments 2019 compared to 2020, for undersupply and oversupply, and for gynecologists do not hold up to a Bonferroni-Holm correction.

Throughout this analysis, no expected cell frequencies were below five, except for surgeons and orthopedists on the one hand and otorhinolaryngologists on the other. For these two comparisons, Fisher’s exact test was applied, but without showing effects statistically different from zero.

The comparison of the overall effects for the simple and extended model can be seen in Table 3.

**Table.3:** LMEM - Comparison of the Overall Effects of the Models

	Simple Model				Extended Model			
Source	N df	D df	F	*p*-Value	N df	D df	F	p-Value
Intercept	1	190.69	1383.61	< .001	1	234.22	1224.95	< .001
Medical specialization	4	191.71	21.86	< .001	4	186.62	23.89	< .001
Year	1	199.45	90.49	< .001	1	169.79	105.12	< .001
Insurance status	1	175.54	48.41	< .001	1	167.14	51.30	< .001
Level of supply	1	193.56	10.55	.001	1	183.87	9.97	.002
Weekday					4	385.56	3.57	.007
Daytime					1	320.15	4.76	.030
Medical specialization * Year					4	166.44	9.24	< .001

The results of the LMEMs for both models are presented in Table 4. Compared to the simple model with an AIC of 1578, the extended model exhibits an AIC of 1551 and can thus display the data more appropriately. Nevertheless, the comparison of the simple with the extended model was applied to examine the robustness of the results. The comparison reveals that, despite controlling for weekday and time of call as well as including an interaction term in the extended model, the main effects are almost the same in both models. Thus, it can be assumed that both models can robustly represent the effects.

**Table.4:** LMEM - Comparison of the Models

	Simple Model			Extended Model		
Parameters	Estimate	SE	p-Value	Estimate	SE	p-Value
Intercept	3.597	.166	< .001	3.394	.188	< .001
Surgeons & Orthopedists	−1.205	.173	< .001	−1.082	.190	< .001
Gynecologists	−.217	.167	.195	−.393	.185	.034
Dermatologists	.046	.219	.834	.211	.240	.382
Otorhinolaryngologists	−.980	.194	< .001	−.673	.212	.002
Year 2020	−.743	.078	< .001	−.750	.180	< .001
PHI	−.435	.063	< .001	−.432	.060	< .001
Oversupply	−.378	.116	.001	−.367	.116	.002
Friday				−.140	.132	.290
Thursday				.078	.086	.363
Wednesday				.305	.113	.008
Tuesday				.258	.111	.020
Afternoon				.168	.077	.030
Year 2020* Surgeons & Orthopedists				−.238	.225	.292
Year 2020* Gynecologists				.619	.227	.007
Year 2020* Dermatologists				−.105	.301	.728
Year 2020* Otorhinolaryngologist				−.584	.250	.021

For the simple model, the overall effect of all independent variables on the logarithmized dependent variable waiting time are statistically significant at the 5% significance level. However, when considering the different medical specializations, it is found that only the effects of surgeons and orthopedists as well as otorhinolaryngologists are statistically significant compared with the reference group of ophthalmologists. Compared with ophthalmologists, patients in surgeons’ and orthopedists’ practices have to face 70.03% (exact computation: 100(e^(− 1.205)^-1)) less waiting time (*p* = <.001, b = − 1.205, SE = .173). For otorhinolaryngologists, the waiting time is 62.47% less than for ophthalmologists (*p* = <.001, b = −.980, SE = .194). The comparison with gynecologists and dermatologists is not statistically different from zero. Considering the year, it appears that COVID-19 reduced waiting time by 7.97 days or 52.43% for patients in 2020 compared to 2019 (*p* = <.001, b = −.743, SE = .078). The insurance status also plays an important role in this model, showing that PHI patients have a 35.27% reduction in waiting time (4.60 days) compared with SHI patients (*p* = <.001, b = −.435 SE = .063). In addition, the simple model reveals that patients in overserved regions receive appointments 31.48% or 3.98 days faster than in underserved regions (*p* = .001, b = −.378, SE = .116).

The directions of the effects all remain the same in the extended model and, moreover, all significant results persist. Again, the overall effects are significant at the 5% level, regardless of the extension of the model.

The fixed effects insurance status and level of supply remain unchanged in the extended model. Hence, PHI patients also receive appointments significantly faster in this design. SHI patients have an increased waiting time by 4.73 days or 35.08% (*p* = <.001, b = −.432 SE = .060). In addition, appointments are scheduled 30.72% faster in overserved than in underserved regions (4.01 days) (*p* = .002, b = −.367, SE = .116).

The fixed effects weekday and daytime of call show overall significant effects. Considering the individual effects, however, it is evident that they are partly not statistically different from zero. Simultaneously, the other results of the model remain stable. This demonstrates that controlling for weekday and daytime does not reduce the influence of the other effects.

Nevertheless, the extension of the model reveals that the effect of year on waiting time is influenced by the different medical specializations. The cross-level interaction shows that the effect of year varies depending on the individual medical specializations. Although the general effect of the year remained nearly stable, with the inclusion of the interaction effect patients received appointments on average 55.60% or 9.05 days faster in 2020 compared to 2019 (*p* = <.001, b = −.812, SE = .079).

With regard to the medical specializations, the comparison between ophthalmologists and three different groups (i.e. surgeons and orthopedists, gynecologists, and otorhinolaryngologists) is statistically significant in this model. On average, patients receive appointments 66.11% faster for surgeons and orthopedists (*p* = <.001, b = − 1.082, SE = .190), 32.50% faster for gynecologists (*p* = .034, b = −.393 SE = .185), and 48.98% faster for otorhinolaryngologists (*p* = .002, b = −.673, SE = .212) compared with ophthalmologists. At the same time, the significant interaction effects for gynecologists and otorhinolaryngologists over time have to be considered. These indicate that waiting times developed differently for the individual specializations. The significant interaction effect for gynecologists shows that this specialization developed differently over time compared to ophthalmologists (*p* = .007, b = .619, SE = .227). On average, patients thus receive appointments at gynecologists more quickly than at ophthalmologists in both years. Yet, compared to ophthalmologists in 2020, the waiting time remains almost stable for gynecologists. The significant negative interaction effect for otorhinolaryngologists (*p* = .021, b = −.584, SE = .250) reveals that in 2020 the waiting times for otorhinolaryngologist appointments decreased more strongly than those for ophthalmologist appointments.

## Discussion

The main purpose of this study was to examine whether the COVID-19 pandemic caused changes in waiting times for outpatient specialist care in Germany. The analysis of the experimental data revealed two major findings. First, in line with the results from the univariate analysis, the multivariate analysis was able to expose, among other effects, that waiting times decreased significantly between 2019 and 2020. Second, however, it was also found that the probability of receiving an appointment at all significantly decreased at the same time. These counteracting effects could be caused by both sides of the physician-patient relation.

On the one hand, it appears desirable that waiting times have decreased in 2020 compared to 2019. On the other hand, the question is what reasons led to this change. These reasons cannot be clearly determined by the analysis.

International studies show a reduction in medical conditions unrelated to COVID-19 in hospitals and emergency departments (ED), for example (acute) myocardial infarction or strokes [30–32]. This can be explained by the fact that overall hospital and ED visits decreased, especially early in the pandemic, leading to an increase in mortality as urgent cases could not be treated in time. The studies conclude that patients no longer visit medical facilities, or visit them too late, because they fear an infection with SARS-CoV-2, resulting in a healthcare access barrier [33–36]. This picture not only emerges for acute cases. There is evidence that patients also used preventive and elective care less [37].

We assume that these results can be applied to our analysis, and that this could be a reason why waiting times were shorter overall in 2020 than in 2019. Patients do not keep their appointments or do not request appointments due to the risk of infection with SARS-CoV-2. Supporting evidence might also be provided by the varying effects of the different medical specializations on waiting time. Compared with the previous year, the waiting times for otorhinolaryngologists decreased the most. In sum, it seems plausible that patients’ higher reluctance to schedule appointments caused the physicians to record an increase in free capacities and to be able to schedule appointments more quickly.

Following this reasoning, it could be assumed that the free capacities are directly linked to a faster allocation of appointments. However, the data analysis shows contradictory results, and patients were even less likely to receive an appointment. This means that even if there is demand, it is served less frequently despite higher free capacities. Hence, the reduced probability of receiving an appointment contradicts the faster allocation of appointments. At the time of data collection, the National Association of Statutory Health Insurance Physicians (NASHIP) had not issued any specific instructions regarding the allocation of appointments (status: April 04, 2020) [38]. Thus, the reduction in supply cannot be attributed to policy interventions. We therefore suggest that reduced supply may also be associated with uncertainty, this time on the provider side. Given that physicians did not receive specific policy instructions and were affected by a completely new situation, non-urgent appointments might have been scheduled less frequently.

Overall, the two main effects suggest that uncertainties due to COVID-19 may have arisen on both the supply and the demand side: Patients go to see physicians less often, and physicians make appointments less frequently. This, however, can cause severe problems when non-urgent cases are left untreated and develop into serious health conditions. The potential risk of focusing only on COVID-19 patients and forgetting about non-COVID-19 patients, such as patients with chronic conditions, has already been pointed out in the literature [26–28, 39]. In the long term, this problem could lead to increased morbidity or mortality in addition to that associated with COVID-19 [27]. This conclusion was also reached in a WHO interim report from 2020, which, in addition to the risk of morbidity and mortality, also indicated that reasons for health service disruption could be attributed to both providers and patients [25].

However, further research is needed to examine the causes of the change in waiting times.

Beside that, the economic consequences of untreated (chronic) diseases must be taken into account. In Germany, an outpatient causes mean costs of €475 per year, whereas an inpatient causes mean costs of €4239 [40]. Therefore, cases that are not treated in outpatient care and result in hospitalizations can have a high financial impact, which is compounded by severe disease progression and mortality.

However, beyond the two main effects studied, our analysis was able to obtain other results that correspond to the findings in existing literature. We evaluated a mean waiting time for SHI of 26.13 and for PHI of 17.37 in 2019, and of 17.09 for SHI and 11.09 for PHI in 2020. The waiting times found by Lungen et al. [21], Heinrich et al. [22], and Werbeck et al. [23] are quite similar. Compared to their studies, our experimental design differs in that we selected practices in over- and underserved regions, called the same practices, contacted all practices as both SHI and PHI patients, and collected our data in the same time period in 2019 and 2020, respectively, to avoid seasonal influences. Nevertheless, in line with their findings, we have also identified unequal access to healthcare, depending on patients’ insurance status. PHI patients receive an appointment significantly faster and more frequently than SHI patients. This effect was also found in studies of inpatient care as well as in studies without an experimental design [14, 15, 19, 20]. Despite the exceptional situation caused by COVID-19, the preferential treatment of PHI patients has not changed. Thus, unequal access to healthcare persists.

In contrast to other studies, we included the level of supply in our analysis to examine whether there are other disparities beside patients’ insurance status. In this context, we found that patients receive appointments more quickly in overserved regions than in underserved ones. Waiting times decreased in both levels of supply between 2019 and 2020. Nevertheless, the difference between the regions remained stable. This shows that, in addition to insurance status, the regions’ level of supply can also constitute a healthcare access barrier. Although we controlled for the weekday and daytime of call in the extended model of the multivariate analysis, all of these results were robust and significant.

Our study has some limitations. One limitation is that only one call was made per insurance status in each year, and that this single call for SHI and PHI patients may not be representative of ordinary practice organization.

Another limitation arises from the fact that the analysis cannot determine a direct effect of COVID-19 on waiting times. To address this issue, consideration was given to the inclusion of COVID-19 incidence in the multivariate analysis. Unfortunately, incidence values are not available in the administrative districts of the practices for the days of calls. Therefore, the overall incidence for the federal state of Bavaria was included on the days of calls. However, the effect of this variable was not statistically different from zero in either model examined. Furthermore, the previously significant effect of year was also no longer statistically different from zero after COVID-19 incidence had been included, and both AIC values worsened. The subsequent examination of the VIF value provided strong evidence of multicollinearity. Therefore, COVID-19 incidence was removed from the models. However, the year dummy can simultaneously be considered a dummy variable for COVID-19.

In addition, a reform was introduced in Germany on May 11, 2019 to ensure that patients receive appointments more quickly (Appointment Service and Supply Act (TSVG)) [41]. Thus, a mixed effect could be present. Given that the reform was introduced with the goal of SHI patients receiving an appointment as quickly as PHI patients [41], however, the study results demonstrate that an unequal distribution still remains. Nevertheless, it can be assumed that the enormous magnitude of the COVID-19 pandemic had a strong impact on the practices. Moreover, our analysis represents only a snapshot at the beginning of the first strong outbreak of COVID-19 in Germany and the results may not be generalizable. Waiting times and the likelihood of receiving an appointment can constantly change during the course of the COVID-19 pandemic. Yet despite this, and in light of the possibility of vaccination, our implications remain. We are still in a state of emergency, and it is not yet foreseeable when medical care will be able to return to a regular schedule. Furthermore, such drastic events as the COVID-19 pandemic can occur again, and both physicians and patients should be suitably prepared for these circumstances. The results show that even in exceptional situations such as a pandemic, regular care should not be disregarded and constant care should be maintained for patients. Sufficient resources must be available for physicians to provide medical care alongside pandemic care. At the same time, patients must be supported in seeking medical care as needed. Only through continuous care can inferior health outcomes be avoided. There is now the chance to learn from such observations for the future, to be able to mitigate medical as well as economic risks in the best possible way.

## Conclusions

The statistical analysis of the data collected in our experiment has practical relevance, as it shows that, in addition to the health risk emerging from the COVID-19 pandemic, regular patient care is also impaired due to the current situation. Although the waiting times for most medical specializations have decreased, the likelihood of receiving an appointment at all has also decreased. Patient uncertainty due to the fear of a potential infection with SARS-CoV-2 could be the cause of free capacities in practices. If appointments are not kept, there is a long-term risk of worsening health status and thus health outcomes. Beside medical risks, this could also have health economic consequences. Although policy decisions are currently concerned with the COVID-19 pandemic, regular care must not be ignored. It has to be ensured that physicians can treat patients safely in their practices. In addition, encouraging patients to continue to seek medical care and arrange appointments should be of interest to public health. To this end, it is particularly important to moderate patients’ fears and uncertainties. This should be ensured by both policymakers and by the healthcare providers themselves. Only if the supply and demand side manage to normalize care, can long-term negative consequences be avoided.

## Data Availability

The datasets used and analyzed in this study are available from the corresponding author on reasonable request.

## Abbreviations

AIC: Akaike information criterion
BASHIP: Bavarian Association of Statutory Health Insurance Physicians
COVID-19: Coronavirus disease 2019
ED: Emergency departments
LMEM: Linear mixed effect model
MAR: Missing at random
NASHIP: National Association of Statutory Health Insurance Physicians
OECD: Organisation for Economic Co-operation and Development
PHI: Private health insurance
SARS-CoV-2: Severe acute respiratory syndrome coronavirus 2
SHI: Statutory health insurance
TSVG: Appointment Service and Supply Act
WHO: World Health Organization
